# Immune Microenvironment Characteristics of Urachal Carcinoma and Its Implications for Prognosis and Immunotherapy

**DOI:** 10.3390/cancers14030615

**Published:** 2022-01-26

**Authors:** Xinke Zhang, Suijing Wang, Run-Cong Nie, Chunhua Qu, Jierong Chen, Yuanzhong Yang, Muyan Cai

**Affiliations:** 1 State Key Laboratory of Oncology in South China, Collaborative Innovation Center for Cancer Medicine, Sun Yat-sen University Cancer Center, Guangzhou 510060, China; zhangxk@sysucc.org.cn (X.Z.); wangsj@sysucc.org.cn (S.W.); nierc@sysucc.org.cn (R.-C.N.); quch1@sysucc.org.cn (C.Q.); chenjr2@sysucc.org.cn (J.C.); 2 Guangdong Provincial Key Laboratory of Orthopedics and Traumatology, Guangzhou 510080, China

**Keywords:** immune microenvironment, urachal carcinoma, prognosis, immunotherapy

## Abstract

**Simple Summary:**

Urachal carcinoma (UrC) is an exceedingly rare tumor and lacks effective treatment. Our study had some important suggestions for targeting programmed cell death-1 (PD-1)/programmed cell death-Ligand 1 (PD-L1) checkpoint in UrC. We fully analyzed the immune microenvironment including intratumoral and peritumoral immune cells, and most of immune cells exerted an immunosuppressive effect; how to reinvigorate immune cells to prevent tumor development would become an important strategy for the treatment of UrC. Tumors with high CD8+ T cell densities also had increasing proportion of PD1 and PD-L1 expression on immune cells, suggesting these partial patients may have developed an activate adaptive immune resistance that might be reversed by treatment of anti-PD-1/PD-L1. No significant difference was found between PD-L1 expression, Mayo stages, and histological type, manifesting that checkpoint inhibitors might be effective for tumors of both early and late stages, as well as with different histological types. Interestingly, we found that the average number of tertiary lymphoid structures (TLS) per slide tended to be higher in tumors with deficient mismatch repair (dMMR) that are promising candidates for immunotherapy, and tumors with higher number of TLS tended to have longer OS and DFS. Increasing CD8+ T cell density was significantly associated with increasing proportion of PD-L1 and PD1 expression on immune cells, and tumors with PD-L1 positive expression on immune cells had significantly increasing proportion of PD1 expression. High peritumoral CD8+ T cell density (>73.7/mm^2^) was significantly associated with worse OS and DFS. Therefore, the number of TLS seems to be considered not only as histopathological characteristics in predicting MMR status of UrC, but also as the prognostic or therapeutic biomarker, and we also provide some important suggestions for targeting PD-1/PD-L1 checkpoint in UrC. UrC immunosuppressive microenvironment would provide deeper understanding between immune cells, in particular CD8+ T cells, and immunosuppression, thereby facilitating discovery of more rational immunotherapeutic strategies.

**Abstract:**

Urachal carcinoma (UrC) is an exceedingly rare tumor and lacks effective treatment. Herein, we characterized an immune microenvironment characteristic of UrC in detail and identified its implications for prognosis and immunotherapy. In total, 37 resections of UrC were stained for CD20, CD3, CD4, CD8, FOXP3, CD68, HLA-DR, CD163, PD1, and PD-L1, as well as mismatch repair protein including MSH2, MSH6, MLH1, and PMS2 by immunohistochemistry. Intratumoral and peritumoral immune cell densities or the proportion of PD1 and PD-L1 expression alongside MSH2, MSH6, MLH1, and PMS2 status were manually evaluated using the whole slide. UrC patients with the number of tertiary lymphoid structures (TLS) per slide tended to be higher in tumors with dMMR (*p* = 0.1919), and tumors with higher number of TLS tended to have longer OS (*p* = 0.0940) and DFS (*p* = 0.0700). High densities of CD3+ T, CD8+ T, and CD68+ cells were significantly associated with worse OS and DFS (both *p*<0.05). Increased intratumoral (*p* = 0.0111) and peritumoral (*p* = 0.0485) CD8+ T cell densities were significantly associated with PD-L1 expression or increasing proportion of PD-L1 expression on immune cells. Similarly, increased intratumoral (*p* = 0.0008) and peritumoral (*p* = 0.063) CD8+ T cell densities were significantly associated with increasing proportion of PD1 expression on immune cells. Tumors with PD-L1 positive expression on immune cells had a significantly increased proportion of PD1 expression (*p* = 0.0121). High peritumoral CD8+ T cell density (>73.7/mm^2^) was significantly associated with worse OS (*p* = 0.0120) and DFS (*p* = 0.00095). The number of TLS seems to be considered not only as histopathological characteristics in predicting MMR status of UrC, but also as a prognostic or therapeutic biomarker, and we also provide some important suggestions for targeting PD-1/PD-L1 checkpoint in UrC.

## 1. Introduction

Urachal cancinoma (UrC) is an extremely rare and highly aggressive tumor, which accounts for 0.35% to 0.70% of bladder cancers [1,2]. Patients with UrC often show a gradually reginal growth and are prone to distant metastasis, thus a large amount of cases with UrC manifest to be at Mayo Stage III or IV [2], and have a poor prognosis. However, so far the mainstreaming therapeutic strategy for UrC remains to be a combination of partial or radical cystectomy with en bloc removal of the umbilical ligament up to umbilicus [3]. Impressively, due to postoperative recurrence and/or metastasis, approximately 21% to 48% of patients still require further adjuvant treatment used in bladder cancer, such as chemotherapy, including cisplatin-based combination therapies (doxorubicin, vinblastine, methotrexate, and gemcitabine) and 5-fluorouracil (FU), which exhibit response rates of 30% to 40%, however, long-standing survival rates remain low [1,4], and radiotherapy plays a limited role in the therapy of UrC as well [5]. Therefore, it is critical to select appropriate treatment strategies to reasonably treat patients with UrC.

In recent years, tumor immunotherapies, such as immune checkpoint inhibitors (ICIs), are promising treatments that have been developed based on the mechanisms of tumor immune escape; which functions by restoring tumor-induced immunosuppression, leading to escape in order to further kill tumor cells [6]. Immunotherapies have transformed the clinical treatment landscape for multiple solid tumors [7,8,9]; however, there are some patients who cannot benefit from immunotherapies due to innate or acquired resistance to them [10]. Therefore, a better understanding of interactions between tumor and intrinsic or adaptive immune response may be helpful for screening the potential beneficial population of effective tumor immunotherapies.

The tumor immune microenvironment, including immune cell infiltration, immune checkpoint expression in tumors, implicates the immunotherapy resistance mechanisms associated with intrinsic or adaptive immune responses [11,12]. Generally, an adaptive immune resistance mechanism is the upregulation of PD-L1 expression on tumor cells or tumor-associated macrophages (TAMs) or dendritic cells induced by inflammatory cytokines from tumor-infiltrating T cells [13]; chemokines or cytokines secreted by these T cells also recruit immunosuppressive M2 macrophages or Tregs or myeloid-derived suppressor cells (MDSCs) into tumors [14,15]. The intrinsic immune resistance mechanism is due to a lack of CD8+ T cells infiltration in tumors or a deficiency of T cell activating signaling pathway, which usually manifests a poor response to PD-1/PD-L1 inhibitors [16]. These studies suggest that the tumor immune microenvironment could significantly affect immunotherapy efficacy. Therefore, in this study, we evaluate the immune microenvironment characteristics of UrC to describe a detailed immune landscape that could provide evidence for the immunotherapeutic efficacy for patients with UrC in the future.

## 2. Materials and Methods

### 2.1. Patients and Tissue Specimens

In our study, we collected 37 samples with UrC patients who primarily underwent their first surgical resection in Department of Pathology, Sun Yat-sen University Cancer Center, from June 2003 to September 2019. The total follow-up period was from the date of diagnosis to that of death or the last date censored if patients remained alive. All the patients did not receive neoadjuvant chemotherapy or radiotherapy. The diagnosis of all samples was reviewed by two experienced pathologists, based on the 2016 WHO criteria for tumor classification, and tumor staging was performed according to the Mayo and Sheldon pathological staging system, which is described as: Stage I, confined to urachus bladder; Stage II, beyond urachus/bladder; Stage III, regional lymph nodes; and Stage IV, distant lymph nodes/metastases. A representative block was selected from every specimen for immunohistochemical (IHC) staining and evaluation.

### 2.2. IHC Staining and Evaluation in UrC

Paraffin blocks were cut into 3-μm sections and stained by IHC according to standard EnVision™ procedure [17,18]. IHC staining of CD20 (OTI4B4, Mouse mAb, ZSGB-BIO, dilution 1:200), CD3 (EP41, Rabbit mAb, ZSGB-BIO, dilution 1:100), CD4 (EP204, Rabbit mAb, ZSGB-BIO, dilution 1:100), CD8 (SP16, Rabbit mAb, ZSGB-BIO, dilution 1:100), FOXP3(221D, Mouse monoclonal, Abcam, dilution 1:50), CD68(KP1, Mouse mAb, ZSGB-BIO, dilution 1:400), HLA-DR (EPR3692, Rabbit mAb, Abcam, dilution 1:250), CD163 (ZM0428, Mouse mAb, ZSGB-BIO, dilution 1:200), PD1 (D4W2J, Rabbit mAb, CST, dilution 1:50), PD-L1 (E1L3N, Rabbit mAb, CST, dilution 1:100), MSH2 (RED2, Rabbit mAb, ZSGB-BIO, dilution 1:100), MSH6 (SP93, Rabbit mAb, Abcam, dilution 1:100), MLH1 (ES05, Mouse mAb, DAKO, dilution 1:100), and PMS2 (EP51, Rabbit mAb, DAKO, dilution 1:100) were performed. The stained sections were observed under microscope and counted in five high-power fields, and the average density of immune cells was calculated as cell counts/mm^2^ for CD20, CD3, CD4, CD8, FOXP3, CD68, HLA-DR, and CD163 in intratumoral and peritumoral stroma, respectively. The percentages of PD-L1 expression in tumor cells and stromal immune cells, alongside with PD-1 positive expression in stromal immune cells were assessed using a semiquantitative score, and the scoring criteria were as follows: each sample tissue harbored an intensity score (I score) from 0–3 (I0-negative expression, I1-weak expression, I2-moderate expression, and I3-strong expression), and percentage score (P score: 0–100%) was obtained according to the percentage of positively stained cells. Any membranous and/or cytoplasmic staining percentage of more than or equal to 1% for PD1 or PD-L1 on immune cells was considered positive, which also included more than or equal to 1% of PD-L1 on tumor cells [19,20,21,22,23]. MMR-status was determined by MLH1, MSH2, MSH6, and PMS2 IHC analysis. Staining of samples in our study were performed with negative controls by replacing the corresponding primary antibodies with PBS during incubation of the slides. We also used normal tonsil tissues as positive controls in the experiments.

### 2.3. Statistical Analysis

The cutoff value of high and low density or proportion in immune cells was determined using X-tile software version 3.6.1 (New Haven, CT, USA). Statistical analyses were performed using GraphPad Prism 8 and SPSS software, version 16.0 (SPSS, Chicago, IL, USA) and R, version 3.6.3 (http://www.r-project.org/, accessed on 29 February 2020). The comparison between groups was analyzed by *t*-test for discrete parameters and χ^2^ test or Fisher’s exact test for categorical parameters. Survival analysis was conducted using Kaplan–Meier method. A two-tailed *p*-value less than 0.05 was defined as statistically significant.

## 3. Results

### 3.1. Clinicopathological Features of Urachal Carcinoma

The clinicopathological features of UrC are shown in Table 1. The median age of patients was 51 years (ranging from 27 to 71 years), and the ratio of male to female is approximately 3:1. Patients presented with different Mayo stages with 16.2% being stage II, 62.2% being stage III, and 21.6% being Stage IV; in total, 28 of 37 patients had local recurrence or distant metastases after first surgical resection. In total, 18 cases received adjuvant chemotherapy, including 11 patients who received Gemcitabine or Capecitabine combined with Cisplatin or Oxaliplatin, 3 cases received Capecitabine combinated with Taxol, 2 patients received Capecitabine combinated with Gemcitabine, and the other 2 patients received Taxol combinated with Cisplatin or 5-FU. Overall, 7 patients presented a stable disease (SD) and 11 patients with a progressive disease (PD). The proportion of enteric, mucinous, and mixed adenocarcinoma accounted for 37.8%, 18.9%, and 43.3% in histological type, respectively. IHC staining showed that 3 of 37 (8.1%) patients were categorized as the dMMR status, and these 3 patients were characterized by Mayo Stage III–IV, and 2 of them belonged to mixed adenocarcinoma and the other one was mucinous carcinoma in histological subtype. In addition, 2 patients had local and distal recurrence in 12 months and 9 months after surgery, thus they died 36 months and 9 months postoperation, respectively. The other case remained as no relapse during the 60-month follow-up. The median follow-up period of 37 patients was 29 months (ranged from 4.0 to 131.0 months).

### 3.2. Quantity Pattern of Immune Cells in Urachal Carcinoma

**Figure 1:**
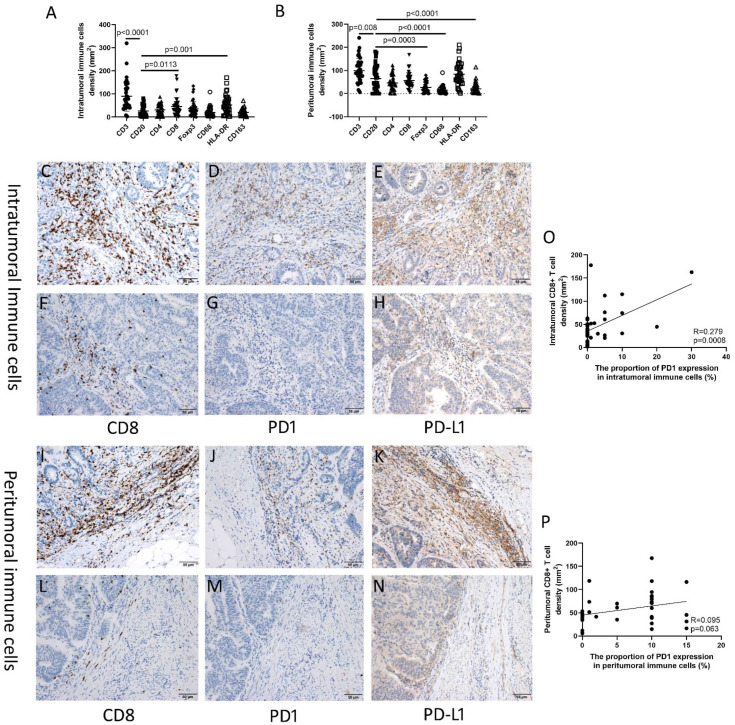
Immune microenvironment of UrC. (**A**) The number of intratumoral immune cells. (**B**) The number of intratumoral immune cells. (**C**–**E**) Relative high density or proportion of CD8, PD1, and PD-L1 expression on immune cells in intratumoral stroma. (**F**–**H**) Relative low density or proportion of CD8, PD1, and PD-L1 expression on immune cells in intratumoral stroma. (**I**–**K**) Relative high density or proportion of CD8, PD1, and PD-L1 expression on immune cells in peritumoral stroma. (**L**–**N**) Relative low density or proportion of CD8, PD1, and PD-L1 expression on immune cells in peritumoral stroma; (**O**,**P**) The association of CD8+ cell density and proportion of PD1 expression on immune cells in intratumoral (**O**) and peritumoral stroma (**P**).

In the intratumoral immune cells that we evaluated across the 37 patients, the median density of CD3+ T cells was 78.6 (range, 2.4–319.0)/mm^2^, CD8+ T cells was 31.8 (1.6–177.8)/mm^2^, CD4+ cells was 28.6 (0.0–87.8)/mm^2^, FoxP3+ cells was 27.4 (0.0–134.4)/mm^2^, CD68+ cells was 14.0 (2.4–108.4)/mm^2^, HLA-DR+ cells was 45.6 (2.6–170.6)/mm^2^, CD163+ cells was 15.0 (1.6–71.2)/mm^2^, and CD20+ B cells was 15.4 (0.0–80.8)/mm^2^ (Figure 1A). The high and low densities of intratumoral immune cells are shown in Supplemental Figure S3. The mean proportion of PD1+ intratumoral immune cells was 3.05% (range, 0.0–30.0%), and the frequency of PD1 positive and negative expression was 40.5% (15/37) and 59.5% (22/37), respectively.

In the peritumoral immune cells, the median density of CD3+ T cells was 91.4 (6.2–241.2)/mm^2^, CD8+ T cells was 45.4 (5.8–167.8)/mm^2^, CD4+ cells was 41.0 (0.0–122.8)/mm^2^, FoxP3+ cells was 17.6 (0.0–79.4)/mm^2^, CD68+ cells was 10.2 (0.0–90.4)/mm^2^, HLA-DR+ cells was 74.2 (1.6–210.4)/mm^2^, CD163+ cells was 10.6 (1.4–114.4)/mm^2^, and CD20+ B cells was 46.0 (0.0–182.0)/mm^2^ (Figure 1B). The high and low densities of peritumoral immune cells are shown in Supplemental Figure S3. The mean proportion of PD1+ immune cells was 5.95%, and the frequency of PD1 positive and negative expression was 67.6% (25/37) and 32.4% (12/37), respectively.

### 3.3. Associations between Immune Characteristics and Clinicopathological Features in Urachal Carcinoma

No significant association was found between the densities of the intratumoral immune cells and gender, age, MMR status, tumor size, vascular invasion, and perineural invasion, respectively. Tumors at Stage IV had significantly higher density of HLA-DR+ cells than those at Stages II and III (*p* = 0.0027), and the densities of CD163+ cells were marginal higher in tumors at Stage IV than those at Stage II and III (*p* = 0.0779). Enteric and mixed adenocarcinoma had a higher density trend of immune cells than mucinous adenocarcinoma, especially CD4+ (*p* = 0.0826) and CD68+ (*p* = 0.1256) cells. In tumors with postoperative relapse, there tended to be higher densities of CD8+ T (*p* = 0.1904), FOXP3+ (*p* = 0.1134), and HLA-DR+ (*p* = 0.1016) cells were observed (Table 1). Tumors at Stage IV had an increasing proportion of PD1 expression compared to those at Stages II and III (*p* = 0.0099) (Supplemental Figure S1).

No significant association was observed between the densities of the peritumoral immune cells and gender, age, tumor size, vascular invasion, Mayo stage, and MMR status, respectively. Tumors with perineural invasion had significantly higher CD3+ T cells (*p* = 0.0447), and a higher CD4+ cells (*p* = 0.0752) trend than those without perineural invasion. In tumors with postoperative relapse, there tended to be higher densities of CD3+ T (*p* = 0.0303) and CD8+ (*p* = 0.0407) cells (Table 2). We also evaluated PD1 expression in 18 patients with recurrence who received adjuvant therapy, and results showed that tumors with postoperative PD had significantly increasing proportion of PD1 expression compared to those with postoperative SD (*p* = 0.0305) (Supplemental Figure S1).

The number of tertiary lymphoid structures (TLS) per slide tended to be higher in tumors with dMMR (*p* = 0.1919).

### 3.4. Associations between Immune Cell PD-L1 Expression and Clinicopathological Features and Immune Characteristics in Urachal Carcinoma

The tumor cells of 36 patients with urachal carcinoma did not have PD-L1 expression and only 1 patient (2.78%) had PD-L1 membranous expression in approximately 1% tumor cells. Immune cell PD-L1 positive and negative proportion was 35.14% (13/37) and 64.86% (24/37), respectively, and tumors with PD-L1 immune cells had significantly higher densities of intratumoral CD3+ T (*p* = 0.0019), CD4+ (*p* = 0.0067), CD8+ T (*p* = 0.0111), and FOXP3+ (*p* = 0.0016) cells and increasing proportion of PD1 expression (*p* = 0.0121) than tumors with PD-L1 negative immune cell. Similarly, tumors with PD-L1 positive immune cells had significantly higher densities of peritumoral CD3+ T (*p* = 0.0266), CD4+ (*p* = 0.0066), and tended to have higher peritumoral CD8+ T (*p* = 0.1485) cells and increasing proportion of PD1 expression (*p* = 0.1550) than tumors with PD-L1 negative immune cell (Table 3). No significant associations were observed between immune cell PD-L1 expression and clinicopathological features (All *p* > 0.05, Table 4).

### 3.5. Association between CD8+ T Cell Density and PD-L1 or PD1 Expression on Immune Cells in Urachal Carcinoma

**Figure 2:**
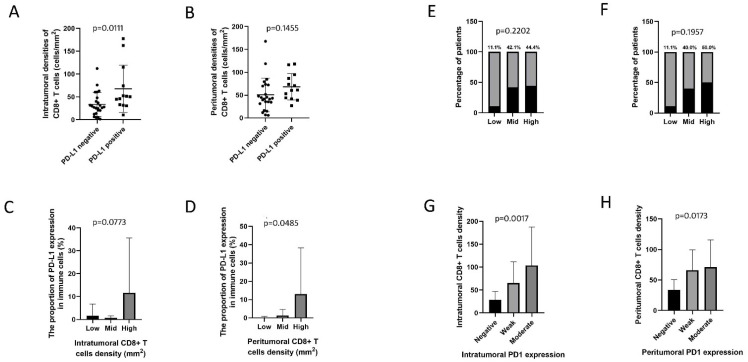
The association between PD-L1 expression and CD8+ T cell density. (**A**) The association between PD-L1 expression and intratumoral CD8+ T cell density. (**B**) The association between PD-L1 expression and peritumoral CD8+ T cell density. (**C**) The association between the percentage of PD-L1 expression and intratumoral CD8+ T cell density as levels by quartiles. (**D**) The association between the percentage of PD-L1 expression and peritumoral CD8+ T cell density as levels by quartiles. (**E**) The association between the percentage of patients with PD-L1 expression and intratumoral CD8+ T cell density as levels by quartiles. (**F**) The association between the percentage of patients with PD-L1 expression and peritumoral CD8+ T cell density as levels by quartiles. (**G**) The association between intensity score of intratumoral PD1 expression and intratumoral CD8+ T cell density as levels by quartiles. (**H**) The association between intensity score of peritumoral PD1 expression and peritumoral CD8+ T cell density as levels by quartiles.

We compared CD8+ T cell density in UrC with or without PD-L1 expression on immune cells. Results showed that intratumoral CD8+ T cell density was significantly higher in tumors with PD-L1 positive immune cells (*p* = 0.0111), which tended to have much higher peritumoral CD8+ T cell density than those with PD-L1 negative immune cells (*p* = 0.1455). Intratumoral CD8+ T cell densities were classified as levels by quartiles: low (1.6–22.9/mm^2^), mid (23.0–56.5/mm^2^), and high (56.6–177.8/mm^2^). There was a marginally significant association between increasing proportion of PD-L1 expression on immune cells and intratumoral CD8+ T cell densities (*p* = 0.0773). Peritumoral CD8+ T cell densities were classified as levels: low (5.8–35.4/mm^2^), mid (35.5–73.6/mm^2^), and high (73.7–167.8/mm^2^). Similarly, increasing proportion of PD-L1 expression on immune cells was significantly linked to increasing peritumoral CD8+ T cell density (*p* = 0.0485). Only 11.1% (1/9) of tumors with low intratumoral and peritumoral CD8+ T cell densities exhibited PD-L1 positive expression on immune cells, 42.1% (8/19) and 44.4% (4/9) of tumors with mid or high intratumoral CD8+ densities, respectively, manifested PD-L1 positive expression on immune cells, suggesting tumors with mid or high intratumoral CD8+ T cell densities tended to have a higher proportion of PD-L1 positive immune cells than those with low intratumoral CD8+ T cell densities (*p* = 0.2202). It was observed that 40.0% (8/20) and 50.0% (4/8) of tumors with mid or high peritumoral CD8+ T cell densities, respectively, exhibited PD-L1 positive immune cells, suggesting tumors with mid or high peritumoral CD8+ T cell densities also tended to have a higher proportion of PD-L1 positive immune cells than those with low CD8+ T cell densities (*p* = 0.1957) (Figure 2).

Results also showed that both the increased intratumoral (*p* = 0.0008) and peritumoral (*p* = 0.063) CD8+ T cell densities were significantly associated with an increasing proportion of PD1 expression on immune cells (Figure 1). The increased intratumoral (*p* = 0.0017) and peritumoral (*p* = 0.0173) CD8+ T cell densities were significantly associated with increasing intensity score of intratumoral and peritumoral PD1 expression on immune cells, respectively (Figure 2).

### 3.6. PD-L1 and PD1 Expression and Immune Cells Densities Can Predict OS and DFS in Urachal Carcinomas

**Figure 3:**
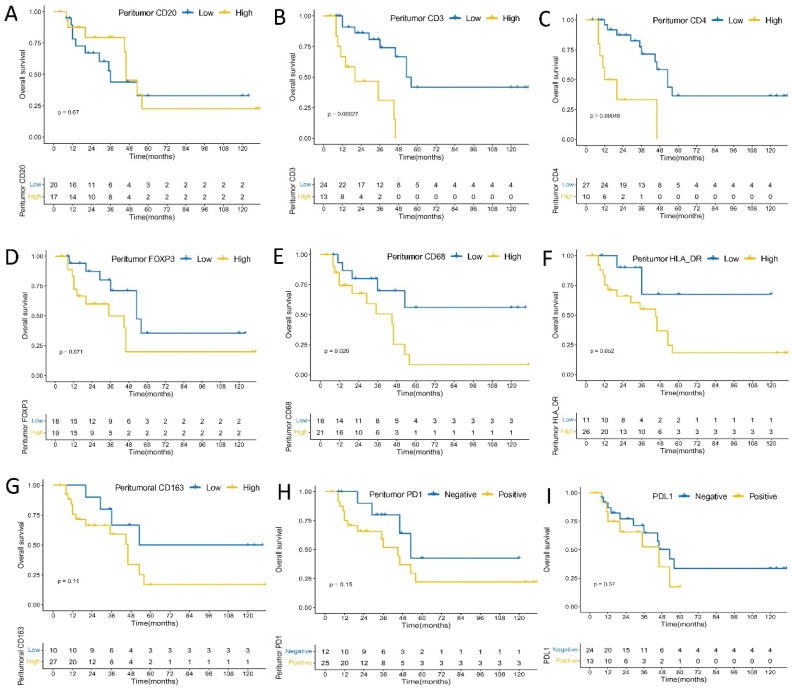
Kaplan–Meier analysis of peritumoral immune cells, PDL1 expression on immune cells and overall survival. (**A**) CD20; (**B**) CD3; (**C**) CD4; (**D**) FOXP3; (**E**) CD68; (**F**) HLA-DR; (**G**) CD163; (**H**) PD1; (**I**) PD-L1 expression.

**Figure 4:**
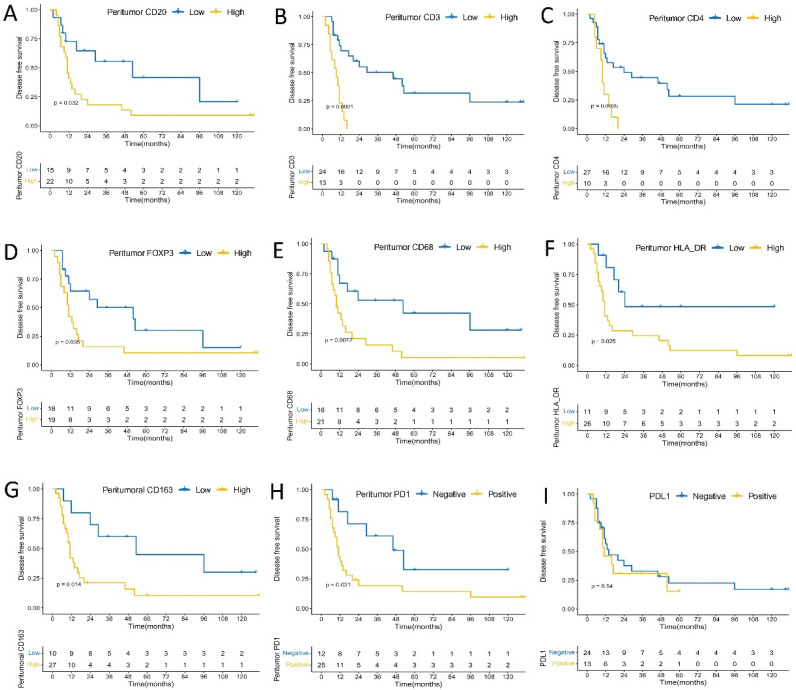
Kaplan–Meier analysis of peritumoral immune cells, PDL1 expression on immune cells and disease free survival. (**A**) CD20; (**B**) CD3; (**C**) CD4; (**D**) FOXP3; (**E**) CD68; (**F**) HLA-DR; (**G**) CD163; (**H**) PD1; (**I**) PD-L1 expression.

**Figure 5:**
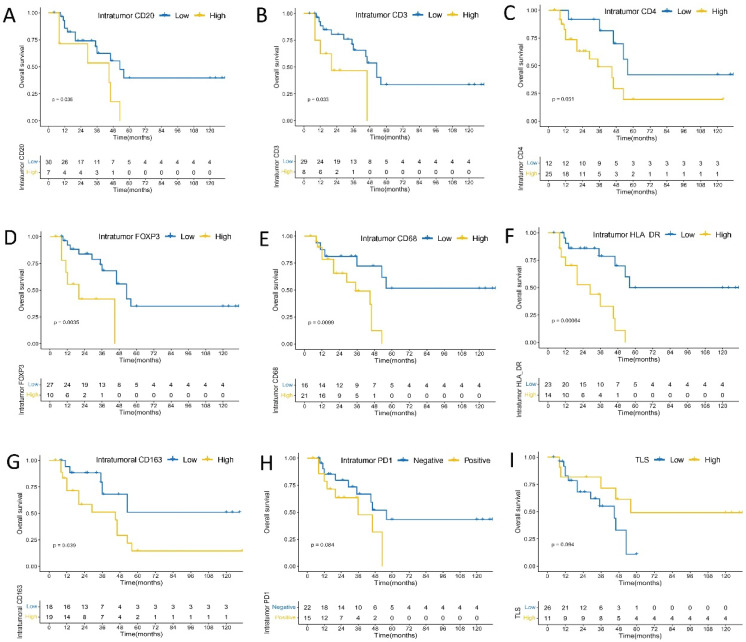
Kaplan–Meier analysis of intratumoral immune cells, TLS and overall survival. (**A**) CD20; (**B**) CD3; (**C**) CD4; (**D**) FOXP3; (**E**) CD68; (**F**) HLA-DR; (**G**) CD163; (**H**) PD1; (**I**) TLS.

**Figure 6:**
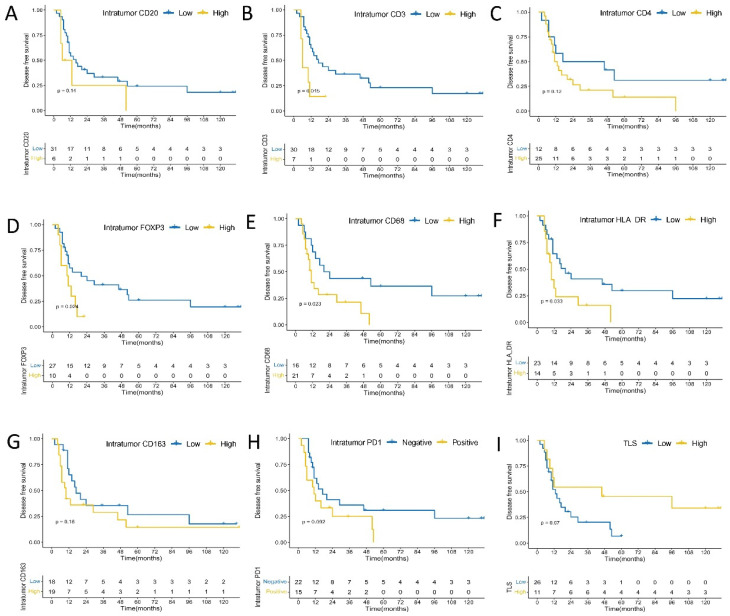
Kaplan–Meier analysis of intratumoral immune cells, TLS and disease free survival. (**A**) CD20; (**B**) CD3; (**C**) CD4; (**D**) FOXP3; (**E**) CD68; (**F**) HLA-DR; (**G**) CD163; (**H**) PD1; (**I**) TLS.

PD-L1 expression on immune cells remained somewhat correlated with worse OS (*p* = 0.3700, Figure 3) and DFS (*p* = 0.5400, Figure 4) in patients with urachal carcinomas, although their association did not reach the level of statistical significance. No significant association was found between intensity score of PD-L1 and DFS (*p* = 0.76) or OS (*p* = 0.40) (Supplemental Figure S2). In intratumoral immune cells, higher densities of CD3+ T (*p* = 0.0330), FOXP3+ (*p* = 0.0035), CD20+ B (*p* = 0.0360), CD68+ (*p* = 0.0099), CD163+ (*p* = 0.0390), and HLA-DR+ (*p* = 0.00064) cells were significantly associated with worse OS, and higher densities of CD4+ cells tended to have the marginal statistic association with poor OS (*p* = 0.0510). In addition, compared to the patients with PD1 negative expression, those with PD1 positive expression on intratumoral immune cells had a marginally significant shorter OS (*p* = 0.0840) (Figure 5). Meanwhile, higher densities of CD3+ T (*p* = 0.0150), FOXP3+ (*p* = 0.0240), CD68+ (*p* = 0.0230), and HLA-DR+ (*p* = 0.0330) cells were significantly correlated with shorter DFS; while higher densities of CD4+ (*p* = 0.1200), CD20+ B (*p* = 0.1400), CD163+ (*p* = 0.1800) cells had the marginal statistic association with poor DFS, and those with PD1 positive expression in intratumoral immune cells had a marginally shorter DFS (*p* = 0.0920) (Figure 6). No significant association was observed between intensity score of intratumoral PD1 and DFS (*p* = 0.24) or OS (*p* = 0.21) (Supplemental Figure S2).

In peritumoral immune cells, higher densities of CD3+ T (*p* = 0.00027), CD4+ (*p* = 0.00048), and CD68+ (*p* = 0.0260) cells were significantly associated with worse OS; higher densities of FOXP3+ (*p* = 0.0710), CD163+ (*p* = 0.1100), and HLA-DR+ (*p* = 0.0520) cells tended to have marginal statistic association with worse OS; tumors with PD1 positive expression in peritumoral immune cells tended to have shorter OS (*p* = 0.1500); and no significant association was found between CD20+ B cells density and OS (*p* = 0.6700) (Figure 3). Higher densities of CD3+ T (*p* < 0.0001), CD4+ (*p* = 0.0035), FOXP3+ (*p* = 0.0350), CD20+ B (*p* = 0.0320), CD68+ (*p* = 0.0072), CD163+ (*p* = 0.0140), and HLA-DR+ (*p* = 0.0250) cells were significantly correlated with shorter DFS. In addition, tumors with PD1 positive expression in peritumoral immune cells had significantly shorter DFS (*p* = 0.0210) (Figure 4). There was a significant association between intensity score of peritumoral PD1 and DFS (*p* = 0.011), but no significant association was found between intensity score of peritumoral PD1 and OS (*p* = 0.22) (Supplemental Figure S2).

Results showed that patients with larger number of TLS per slide tended to have longer OS (*p* = 0.0940) (Figure 5) and DFS (*p* = 0.0700) (Figure 6).

**Figure 7:**
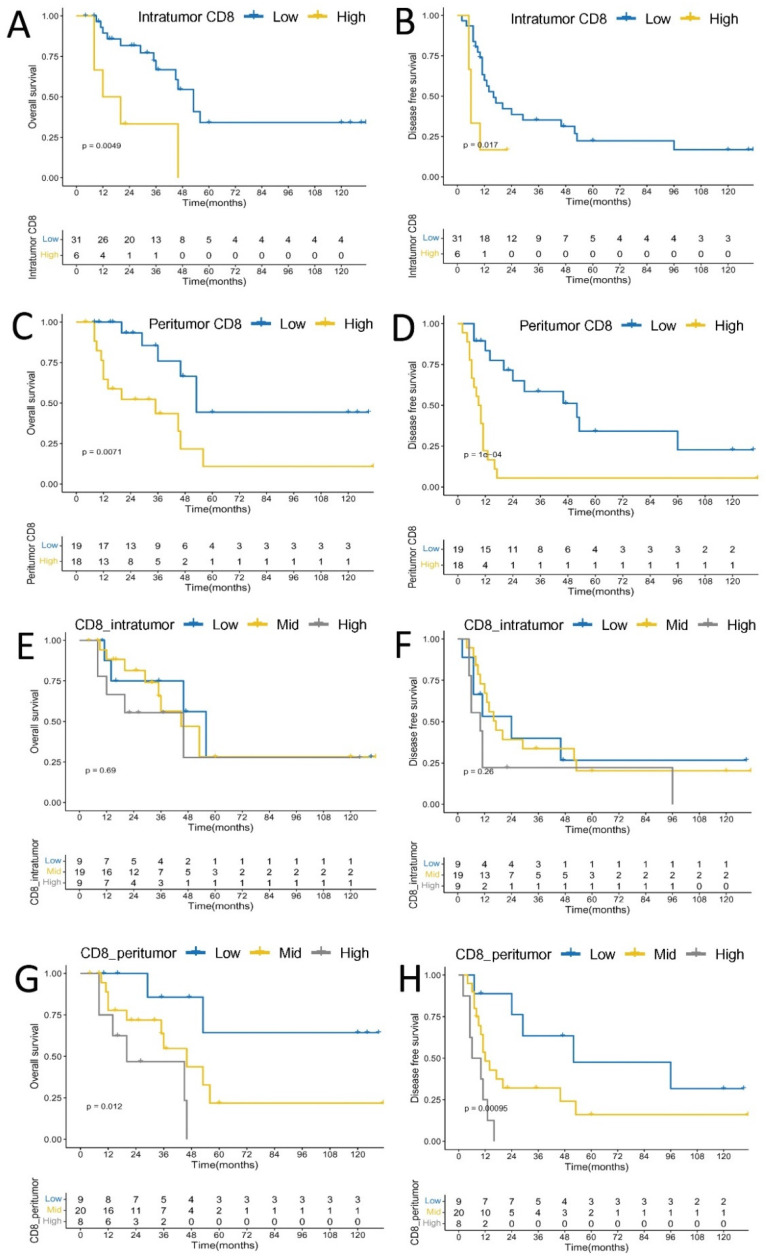
Kaplan–Meier analysis of CD8+ T cell density and overall survival. (**A**) Intratumoral CD8; (**C**) Peritumoral CD8; (**E**) Intratumoral CD8 as levels by quartiles; (**G**) Peritumoral CD8 as levels by quartiles; Kaplan–Meier analysis of CD8+ T cells density and disease free survival. (**B**) Intratumoral CD8; (**D**) Peritumoral CD8; (**F**) Intratumoral CD8 as levels by quartiles; (**H**) Peritumoral CD8 as levels by quartiles.

Finally, intratumoral higher density of CD8+ T cell was significantly associated with worse OS (*p* = 0.0049) and DFS (*p* = 0.0170), respectively, and peritumoral higher density of CD8+ T cell was also significantly linked with worse OS (*p* = 0.0071) and DFS (*p* = 0.0001), respectively. Tumors with high peritumoral CD8+ T cell densities showed worse OS (*p* = 0.0120) and DFS (*p* = 0.00095) than those with low or mid peritumoral CD8+ T cell densities, while there was no significant statistic difference between intratumoral CD8+ T cell densities classified as levels by quartiles and OS (*p* = 0.6900) and DFS (*p* = 0.2600) (Figure 7).

## 4. Discussion

Immune checkpoint inhibitors have been suggested to be effective for cancer therapy, especially for solid tumor with dMMR [24]. In this study, 8.1% of UrC patients were categorized as dMMR based on immunohistochemical staining, and these patients were characterized to be at Mayo Stage III or IV, which is consistent with prior studies that reported that the proportion of patients with dMMR ranged from 0 to 16.7%, mostly at advanced stages [25,26]. In addition, the average number of TLS per slide tended to be higher in tumors with dMMR that are promising candidates for immunotherapy, and tended to have longer OS or DFS, which is in line with the recent study in colorectal cancer [27,28]. Therefore, we speculated that the number of TLS is not only considered as a histopathological characteristic in predicting the MMR status of UrC, but also as the prognostic or therapeutic biomarker.

Grasping the nature of cross-talk between tumor cells and the neighboring immune cells would enable the selection of optimal therapeutics by targeting multiple components of the TME, thereby improving patient prognosis [29]. In intratumoral and peritumoral immune cells, CD3+ T cells are the most prevalent immune cells, and tumor-infiltrating T cells play critical roles in mediating immune escape and anti-tumor immune response [30,31]. CD8+ T cells are the predominant gradient of total T cells both in intratumoral and peritumoral immune cells. In this study, a high density of CD8+ T cells was found to be associated with worse prognosis of UrC, which is in agreement with prior study on gastric adenocarcinomas [20]. The current study demonstrated that different CD8+ T cell subpopulations play different roles within the tumor microenvironment; for example, dysfunctional CD8+ T cells could be linked to tumor progression and poor prognosis, which account for 5~80% of the total infiltrating T cells [32,33]. In addition, we also found that FOXP3+ cell density was slightly higher than that of CD4+ cells in intratumoral immune cells, suggesting FOXP3+ cells could be the part of CD8+ T cells, and FOXP3+ CD8+ T cells subpopulation may contribute to UrC immune escape and disease progression, which have been described in hepatocellular carcinoma [34]. Moreover, previous studies showed that high HLA-DR+ cell density is positively correlated with poor clinical outcomes, and our results also show that the density of HLA-DR+ cells was much more than that of CD68+ cells, suggesting that HLA-DR could express on CD4+ T, CD8+ T, or immature DC cells other than CD68+ cells [35,36,37,38]. Additionally, previous reports showed that increased frequency of CD4+ HLA-DR+ T or HLA-DR+ CD8+ T cells is associated with disease progression in several tumors [35,36,39]. However, high CD68+ HLA-DR+ macrophages are associated with better prognosis in melanoma [37]. Therefore, we speculated that HLA-DR+ cells in intratumoral and peritumoral immune cells of UrC could more likely represent CD4+ or CD8+ T cells belonging to immunosuppressive subtype and immature DC that have a reduced capacity to stimulate T-cells, which somewhat underscores the importance of inefficient antigen presentation as a mechanism for tumor evasion. These findings suggest that intratumoral and peritumoral immune cells, in particular T cells in UrC exert the immunosuppressive effect and how to reinvigorate them to prevent tumor development would become an important strategy for the treatment of UrC.

Finally, no significant difference was found between PD-L1 expression on immune cells and Mayo stages, histological type, manifesting that checkpoint inhibitors might be effective for tumors of both early and late stages, as well as with different histological types. Our study showed that 2.78% and 35.14% of UrC showed PD-L1 expression on tumor and immune cells, respectively, suggesting PD-L1 expression on immune cells might also be associated with the immunosuppressive mechanism beyond PD-L1 expression on tumor cells [40]. PD-L1 expression on immune cells tended to have worse OS and DFS in UrC patients, which has been reported previously in various tumors [20,41,42]. The possible reason for this is that PD-L1 expression on host immune cells can reduce T cell immunity, leading to tumor progression and poor prognosis [43]. Therefore, the expression of PD-L1 might also be a potential predictor and therapeutic target for UrC. Simultaneously, high densities of intratumoral and peritumoral CD8+ T cells are related to PD-L1 positive expression on immune cells, which can be explained by the accumulation of CD8+ T cells in TME, where they could stimulate PD-L1 expression by releasing specific factors [44]. In this study, nearly half of patients with PD-L1 expression on immune cells have high CD8+ T cells density, which somewhat underscores the close association between CD8+ T cells and PD-L1 expression. High CD8+ T cell density is also associated with an increasing proportion of PD1 and PD-L1 expression, suggesting an increasing proportion of PD1 expression cells might cause exhaustion of CD8+ T cells when PD1 interacts with PD-L1 [45]. However, tumors with PD-L1 positive expression in immune cells had a significantly increased proportion of PD1 expression, indicating that these patients may have developed an adaptive immune resistance by PD1/PD-L1 signal pathway, thus immunosuppressive effect may be restored by administration of anti-PD-1/PD-L1. Therefore, we speculated that the evidence obtained from checkpoint inhibitors applied in UrC is based on the close association between high CD8+ T cells density and increasing expression of PD-L1 and PD1. Meanwhile, PD-L1 expression on immune cells might potentiate enhanced immunosuppression by impairing the secreting cytokines function of CD8+ T cells, which is associated with tumor progression and poor prognosis [46], and consistent with our result that tumors with high densities of peritumoral CD8+ T cells had a worse prognosis than those with low or mid densities. Similar findings have been reported in hepatocellular carcinoma [47] and colon cancer [48]. However, the relationship between CD8+ T cells and immunosuppression via enhanced PD-L1 expression remains to be further investigated in UrC. In addition, our results showed that PD1 intensity score gradually increased as the density of CD8+ T cells increased, and tumors with increasing PD1 intensity score had worse prognosis, indicating that expression intensity of PD1 might be closely associated with CD8+ T cell exhaustion, and how to downregulate PD1 to enhance antitumor immunity by improving T cells exhaustion [49] might become an important therapeutic regime in UrC. However, more detailed characteristics of the UrC immune microenvironment would provide deeper understanding between CD8+ T cells and immunosuppression to find more rational immunotherapeutic strategies.

In conclusion, the number of TLS seems to be considered not only as histopathological characteristics in predicting MMR status of UrC, but also as the prognostic or therapeutic biomarker. More interestingly, we fully analyzed the immune microenvironment, in which most of immune cells exerted immunosuppressive effect, which might provide important suggestions for targeting PD-1/PD-L1 checkpoint in UrC.

## 5. Conclusions

The number of TLS seems to be considered not only as histopathological characteristics in predicting MMR status of UrC, but also as a prognostic or therapeutic biomarker. More interestingly, we fully analyzed the immune microenvironment, in which most of immune cells exerted immunosuppressive effect, which might provide important suggestions for targeting PD-1/PD-L1 checkpoint in UrC.

## Figures and Tables

**Table 1:** Correlation between the intratumoral immune characteristics and clinicopathological variables in UrC patients.

Variables	Intratumoral Immune Characteristics (Cells/mm^2^)																
	N	CD20	*p* Value	CD3	*p* Value	CD 4	*p* Value	CD 8	*p* Value	FOXP3	*p* Value	CD 68	*p* Value	HLA-DR	*p* Value	CD163	*p* Value
Gender			0.3615		0.3525		0.3325		0.3275		0.4658		0.9666		0.2176		0.5293
Female	9	19.69		73.31		37.24		35.58		29.60		19.82		36.22		16.24	
Male	28	27.74		97.68		28.38		51.53		39.01		20.17		56.53		19.96	
Age ^(a)^			0.5668		0.6604		0.2749		0.7518		0.8497		0.1799		0.9187		0.6375
≤51	20	23.87		85.76		27.46		47.58		36.70		24.27		52.95		18.82	
>51	17	28.29		95.27		36.06		43.32		34.68		15.39		51.55		21.20	
Tumor size ^(b)^			0.9702		0.4933		0.9194		0.4649		0.5982		0.1971		0.1251		0.0722
≤4 cm	20	25.77		83.33		31.78		41.11		33.20		16.26		42.82		15.83	
>4 cm	17	26.06		98.13		30.98		50.93		38.80		24.81		63.47		24.72	
Vascular invasion			0.9589		0.5833		0.8441		0.2302		0.3313		0.0611		0.2367		0.6254
No	30	25.81		82.87		31.79		41.77		33.30		17.24		48.45		19.32	
Yes	7	26.31		102.4		29.80		62.14		46.37		32.83		68.86		22.46	
Perineural invasion			0.3691		0.2339		0.1316		0.4042		0.0532		0.2422		0.1397		0.5096
No	21	22.90		79.02		26.29		40.75		27.06		16.81		43.65		18.47	
Yes	16	29.85		104.7		38.14		52.01		47.21		24.63		63.68		21.18	
Mayo stage			0.2493		0.4169		0.6842		0.1949		0.5363		0.1201		0.0027		0.0779
II	6	21.90		63.37		27.27		29.57		23.07		15.77		42.07		21.08	
III	23	22.57		90.18		30.32		42.51		36.99		18.15		40.61		16.03	
IV	8	37.90		110.1		37.65		6.60		41.80		29.38		93.63		29.68	
Histological type			0.8698		0.9488		0.0826		0.2879		0.1256		0.5743		0.3572		
Enteric	14	25.76		98.96		33.77		55.41		39.97		23.86		50.46		21.50	
Mucinous	7	21.97		52.00		13.89		23.97		18.63		6.429		39.94		12.49	
Mixed	16	27.83		99.09		37.01		46.53		39.60		23.00		59.34		21.78	
Relapse			0.4716		0.1308		0.8501		0.1904		0.1134		0.4015		0.1016		0.9248
No	9	21.02		62.31		29.69		30.29		21.22		15.27		32.98		20.33	
Yes	28	27.47		99.01		31.96		50.55		40.145		21.77		58.52		19.78	
MMR status ^(c)^			0.5048		0.4016		0.8173		0.4978		0.1047		0.8180		0.9800		0.7490
pMMR	34	26.66		92.78		31.68		46.97		38.28		20.42		52.36		20.15	
dMMR	3	17.27		60.07		28.33		30.33		7.333		17.60		51.73		17.20	

^(a)^: median age; ^(b)^: median tumor size; ^(c)^**:** Mismatch repair status (p/dMMR: proficient/deficient MMR).

**Table 2:** Correlation between the peritumoral immune characteristics and clinicopathological variables in UrC patients.

Variables	Peritumoral Immune Characteristics (Cells/mm^2^)																
	N	CD20	*p* Value	CD3	*p* Value	CD 4	*p* Value	CD 8	*p* Value	FOXP3	*p* Value	CD 68	*p* Value	HLA-DR	*p* Value	CD163	*p* Value
Gender			0.4772		0.6944		0.8820		0.8028		0.5933		0.5357		0.3430		0.7074
Female	9	53.29		92.38		45.00		54.64		23.36		14.87		68.71		15.84	
Male	28	69.80		100.8		46.99		58.13		28.35		12.27		88.32		18.32	
Age ^(a)^			0.7648		0.4899		0.2456		0.7843		0.1532		0.4394		0.3557		0.1000
≤51	20	67.49		105.5		21.91		55.24		31.53		13.20		91.52		15.27	
>51	17	61.71		93.39		39.53		58.46		20.47		17.47		75.75		27.60	
Tumor size ^(b)^			0.3094		0.6421		0.5250		0.5004		0.8135		0.3181		0.8818		0.5086
≤4cm	20	73.80		103.7		47.25		60.35		27.30		12.63		83.10		18.62	
>4cm	17	54.28		95.44		44.84		52.45		25.45		18.14		85.65		23.66	
Vascular invasion			0.5180		0.9274		0.3129		0.3571		0.8195		0.2606		0.2503		0..2391
No	30	67.83		99.53		43.51		54.13		26.88		16.65		88.98		23.07	
Yes	7	51.97		101.6		57.43		67.83		24.60		8.771		64.10		11.74	
Perineural invasion			0.5953		0.1594		0.0447		0.0752		0.1600		0.7878		0.3843		0.9357
No	21	60.37		89.13		36.88		47.82		21.71		14.51		77.81		20.67	
Yes	16	70.69		114.1		58.30		68.40		32.66		16.01		92.76		21.29	
Mayo stage			0.7321		0.4896		0.4054		0.2331		0.7579		0.5021		0.6917		0.1014
II	6	48.71		76.47		34.07		41.83		22.27		22.50		69.70		33.67	
III	23	66.69		102.9		45.28		54.84		28.71		13.57		89.43		17.26	
IV	8	72.00		109.1		57.68		73.28		23.08		14.32		80.39		20.93	
Histological type			0.1502		0.6711		0.1870		0.1086		0.2672		0.0458		0.5277		0.1014
Enteric	14	86.00		109.9		44.64		71.86		34.33		23.36		96.40		31.10	
Mucinous	7	67.49		90.43		28.40		42.91		24.71		6.871		80.60		15.60	
Mixed	16	45.15		95.36		55.21		49.51		20.55		11.61		75.27		14.38	
Relapse			0.2220		0.0303		0.2639		0.0407		0.2287		0.1748		0.3044		0.6097
No	9	44.29		67.00		35.51		36.18		18.22		8.622		68.89		17.51	
Yes	28	71.44		110.5		49.56		63.32		29.09		17.62		89.22		22.04	
MMR status ^(c)^			0.7228		0.5314		0.6937		0.8005		0.1324		0.9010		0.9762		0.9256
pMMR	34	63.82		101.6		46.78		57.16		28.17		15.26		84.20		20.83	
dMMR	3	76.33		81.20		38.99		51.73		6.933		14.00		85.13		22.13	

^(a)^: median age; ^(b)^: median tumor size; ^(c)^**:** Mismatch repair status (p/dMMR: proficient/deficient MMR).

**Table 3:** Correlation between the PDL1 expression in immune cells and immune characteristics in UrC.

Variables (Mean)	PD-L1 Expression in Immune Cells			
	Negative	Positive	*t*-Test	*p* Value
CD20 density (cells/mm^2^)				
Intratumor	21.66	33.74	1.557	0.1286
Peritumor	69.55	56.12	0.6740	0.5048
CD3 density (cells/mm^2^)				
Intratumor	67.04	132.8	3.365	0.0019
Peritumor	85.88	125.8	2.315	0.0266
Intraepithelial	3.525	4.615	0.3888	0.6998
CD4 density (cells/mm^2^)				
Intratumor	23.91	45.26	2.885	0.0067
Peritumor	35.83	65.18	2.886	0.0066
Intraepithelial	0.1583	0.3538	0.5814	0.5646
CD8 density (cells/mm^2^)				
Intratumor	33.63	67.77	2.682	0.0111
Peritumor	50.53	68.14	1.489	0.1485
Intraepithelial	2.825	3.692	0.3728	0.7115
FOXP3 density (cells/mm^2^)				
Intratumor	24.30	56.95	3.417	0.0016
Peritumor	23.77	31.40	0.9493	0.3490
CD68 density (cells/mm^2^)				
Intratumor	16.13	27.69	1.733	0.0919
Peritumor	13.72	17.82	0.7164	0.4785
HLA-DR ^(a)^ density (cells/mm^2^)				
Intratumor	45.29	65.26	1.448	0.1564
Peritumor	86.43	80.29	0.3454	0.7319
CD163 density (cells/mm^2^)				
Intratumor	18.84	21.89	0.5846	0.5626
Peritumor	18.78	24.91	0.7796	0.4409
The proportion of PD1 expression (%)				
Intratumor	1.208	6.462	2.646	0.0121
Peritumor	5.000	7.692	1.453	0.1550
TLS ^(b)^				
The number of per slide	1.453	0.4856	2.907	0.0063

^(a)^: human leukocyte antigen-DR; ^(b)^: tertiary lymphoid structures.

**Table 4:** Correlation between the PD-L1 expression in immune cells and clinicopathological variables in UrC.

Variables	PD-L1 Expression in Immune Cells			
	N	Negative	Positive	*p* Value
Gender				0.8964
Female	9	6(66.67%)	3(33.33%)	
Male	28	18(64.29%)	10(35.71%)	
Age ^(a)^				0.4779
≤51	20	14(70.00%)	6(30.00%)	
>51	17	10(58.82%)	7(41.18%)	
Tumor size ^(b)^				0.9851
≤4 cm	20	13(65.00%)	7(35.00%)	
>4 cm	17	11(64.71%)	6(35.29%)	
Vascular invasion				0.1756
No	30	21(70.00%)	9(30.00%)	
Yes	7	3(42.86%)	4(57.14%)	
Perineural invasion				0.6657
No	21	13(61.90%)	8(38.10%)	
Yes	16	11(68.75%)	5(31.25%)	
Histological type				0.2420
Enteric	14	11(53.6%)	3(46.4%)	
Mucinous	7	5(71.43%)	2(28.57%)	
Mixed	16	8(50.00%)	8(50.00%)	
Mayo stage				0.6044
II	6	4(66.67%)	2(33.33%)	
III	23	16(69.57%)	7(30.43%)	
IV	8	4(50.00%)	4(50.00%)	
Relapse				0.8964
No	9	6(66.67%)	3(33.33%)	
Yes	28	18(64.29%)	10(35.71%)	
MMR status ^(c)^				0.9456
pMMR	34	22(64.71%)	12(35.29%)	
dMMR	3	2(66.67%)	1(33.33%)	
Therapeutic efficacy				0.7829
SD	7	4(57.14%)	3(42.86%)	
PD	11	7(63.64%)	4(36.36%)	

^(a)^: median age; ^(b)^: median tumor size; ^(c)^: Mismatch repair status (p/dMMR: proficient/deficient MMR).

## Data Availability

The data presented in this study are available on request from the corresponding author.

## Supplementary Materials

The following supporting information can be downloaded at: https://www.mdpi.com/article/10.3390/cancers14030615/s1, Figure S1: The association between the proportions of PD1 expression in intratumoral immune cells and (A) Mayo stage, (B) relapse or not, (C) histological type, (D) MMR status, and (E) therapeutic efficacy after operation, and that between peritumoral immune cells and (F) Mayo stage, (G) relapse or not, (H) histological type, (I) MMR status, and (J) therapeutic efficacy after operation, and the association between the number of TLS per slide and (K) Mayo stage, (L) relapse or not, (M) histological type, (N) MMR status, and (O) therapeutic efficacy after operation; Figure S2: Kaplan–Meier analysis of PD1, PD-L1 expression intensities and disease free survival, overall survival. (A) intratumoral PD1 expression intensity and overall survival; (B) intratumoral PD1 expression intensity and disease free survival; (C) peritumoral PD1 expression intensity and overall survival; (D) peritumoral PD1 expression intensity and disease free survival; (E) PD-L1 expression intensity and overall survival; (F) PD-L1 expression intensity and disease free survival; Figure S3: The high and low densities of intratumoral and peritumoral immune cells in UrC (200×). (A–D) CD20; (E–H) CD3; (I–L) CD4; (M–P) FOXP3; (Q–T) CD68; (U–X) CD163; (Y–AB) HLA-DR; (AC) Tumor PD-L1 positive; (AD) Tumor PD-L1 negative; (AE) The presence of TLS; (AF) The absence of TLS.
