# Supplementary material for: Immune Microenvironment Characteristics of Urachal Carcinoma and Its Implications for Prognosis and Immunotherapy
Source: Cancers (Basel). 2022 Jan 26;14(3):615. doi: 10.3390/cancers14030615 (PMC8833550; doi:10.3390/cancers14030615)
Supplement: Supplementary file 1 [file cancers-14-00615-s001.zip › cancers-1495895-supplementary.pdf]

Supplementary Materials:

Immune Microenvironment Characteristics of Urachal Carcinoma and Its Implications for Prognosis and Immunotherapy

Xinke Zhang [1,*,†], Suijing Wang [1,†], Run-cong Nie [1], Chunhua Qu [1], Jierong Chen [1], Yuanzhong Yang [1,*] and Muyan Cai [1,2,*]

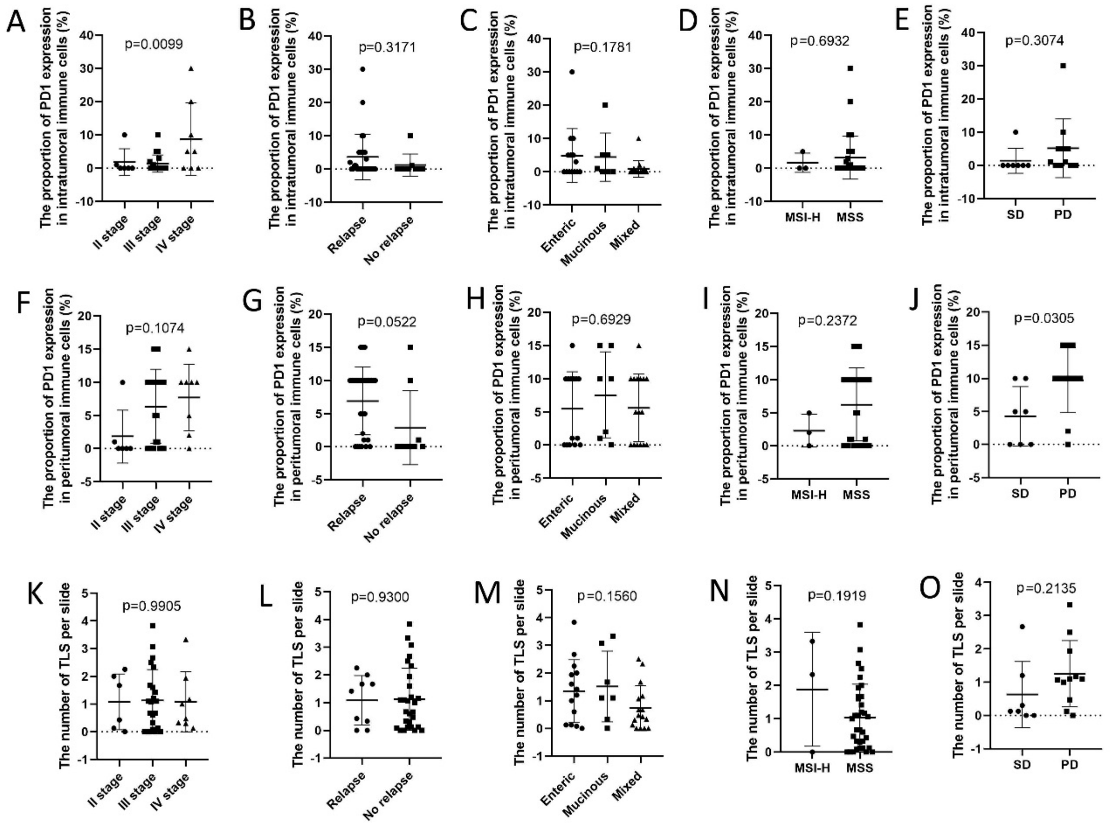

Figure S1: The association between the proportions of PD1 expression in intratumoral immune cells and (**A**) Mayo stage, (**B**) relapse or not, (**C**) histological type, (**D**) MMR status, and (**E**) thera-peutic efficacy after operation, and that between peritumoral immune cells and (**F**) Mayo stage, (**G**) relapse or not, (**H**) histological type, (**I**) MMR status, and (**J**) therapeutic efficacy after opera-tion, and the association between the number of TLS per slide and (**K**) Mayo stage, (**L**) relapse or not, (**M**) histological type, (**N**) MMR status, and (**O**) therapeutic efficacy after operation.

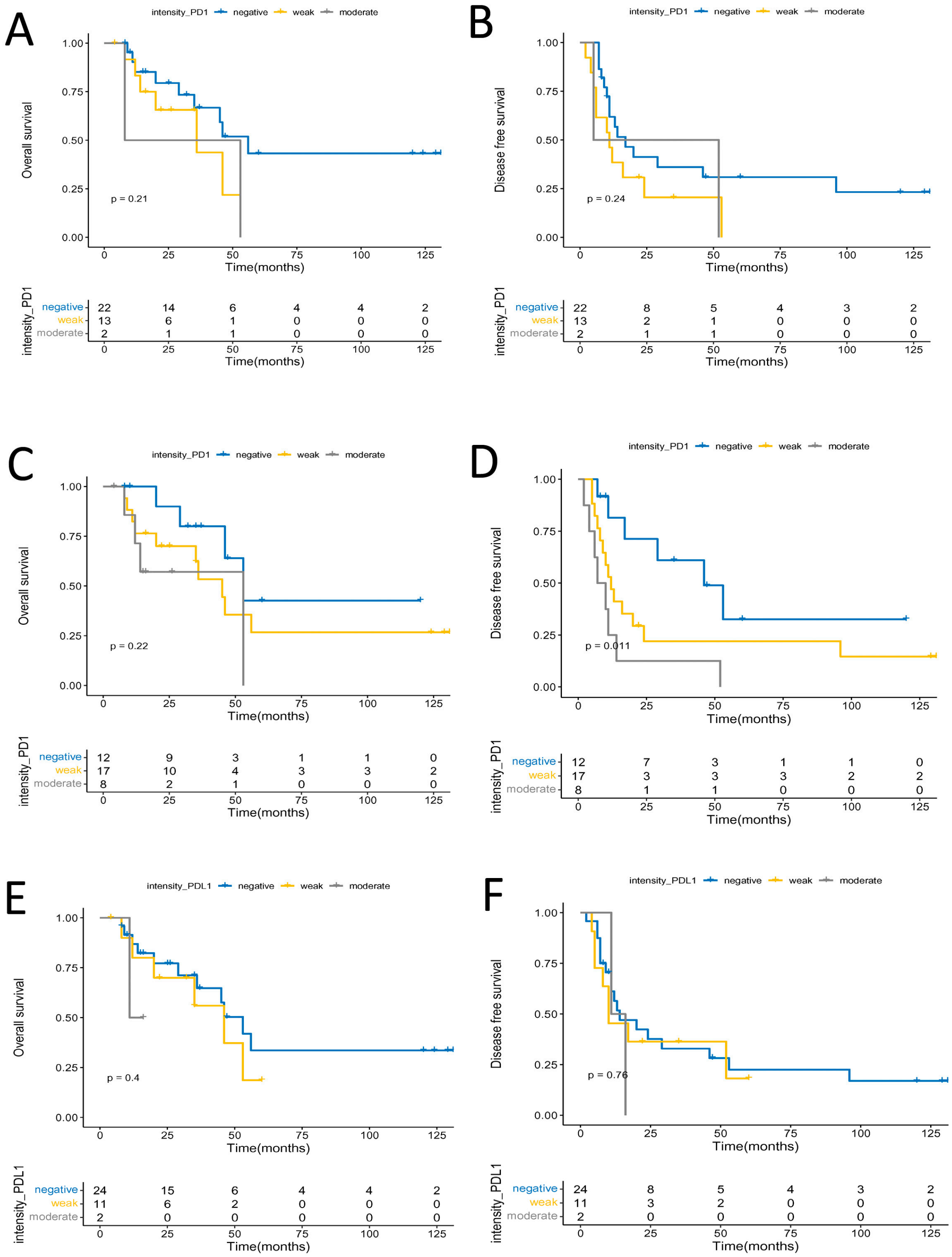

Figure S2: Kaplan–Meier analysis of PD1, PD-L1 expression intensities and disease free survival, overall survival. (**A**) intratumoral PD1 expression intensity and overall survival; (**B**) intratumoral PD1 expression intensity and disease free survival; (**C**) peritumoral PD1 expression intensity and overall survival; (**D**) peritumoral PD1 expression intensity and disease free survival; (**E**) PD-L1 expression intensity and overall survival; (**F**) PD-L1 expression intensity and disease free sur-vival.

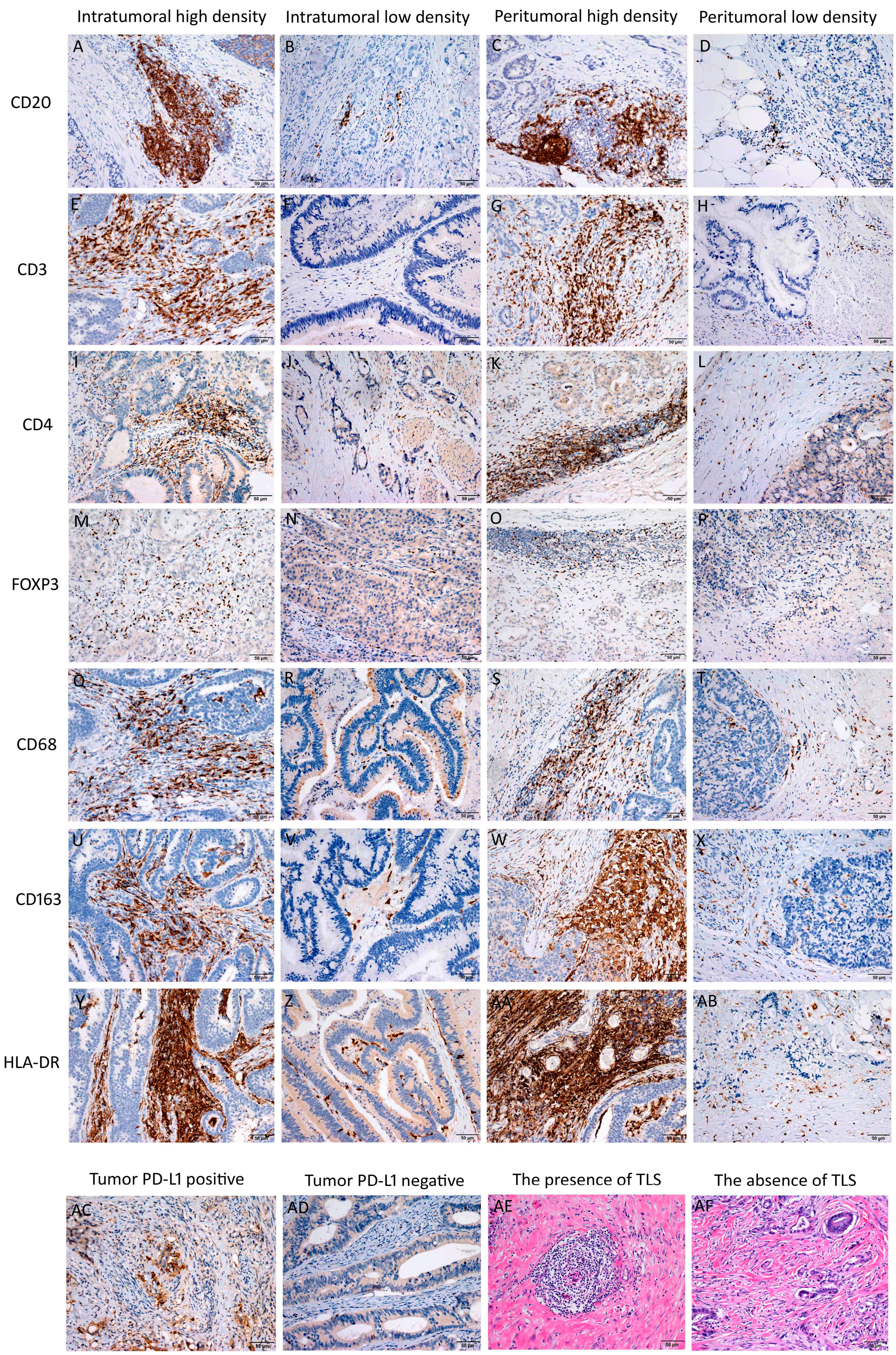

Figure S3: The high and low densities of intratumoral and peritumoral immune cells in UrC (200X). (**A**–**D**) CD20; (**E**–**H**) CD3; (**I**–**L**) CD4; (**M**–**P**) FOXP3; (**Q**–**T**) CD68; (**U**–**X**) CD163; (**Y**–**AB**) HLA–DR; (**AC**) Tumor PD–L1 positive; (**AD**) Tumor PD–L1 negative; (**AE**) The presence of TLS; (**AF**) The absence of TLS.
